# Synchronous Primary Malignancies: Incidental Detection of Ascending Colon Adenocarcinoma During Staging of Invasive Ductal Carcinoma of the Breast

**DOI:** 10.1155/crom/7164628

**Published:** 2025-03-11

**Authors:** Salif Balde, Ulrich Igor Mbessoh Kengne, Jaafar Ibn Abou Talib Thiam, Joël Gabin Konlack Mekontso, Sokhna Diop Niang, Amacoumba Fall, Mamadou Ndiaye, Gorgui Sarr, Etienne Tossou Zoure, Mamadou Sow, Sidy Ka

**Affiliations:** ^1^Department of Oncology, Dalal Jamm National Hospital Center, Dakar, Senegal; ^2^Department of Surgery and Specialties, Faculty of Medicine, Pharmacy and Odontostomatology, Cheikh Anta Diop University, Dakar, Senegal; ^3^Department of Internal Medicine, New York City Health and Hospitals South Brooklyn Health, Brooklyn, New York, USA; ^4^Department of Internal Medicine, Dalal Jamm National Hospital Center, Dakar, Senegal

**Keywords:** breast cancer, colon cancer, multidisciplinary team meeting, multiple primary malignant neoplasms, synchronous primary malignancies

## Abstract

Multiple primary malignant neoplasms (MPMNs) are defined as two or more distinct tumors in the same individual. Synchronous breast and colon cancers are infrequent and present management challenges due to the lack of standardized guidelines. We report a 73-year-old woman presenting with a right breast mass, subsequently diagnosed as Grade 2 invasive ductal carcinoma. Staging CT incidentally revealed right colon wall thickening, and colonoscopy with biopsy confirmed moderately differentiated invasive adenocarcinoma. Following neoadjuvant chemotherapy, she underwent simultaneous radical mastectomy with axillary lymph node dissection and right hemicolectomy. Postoperative recovery was uneventful. Adjuvant chemoradiation was administered per multidisciplinary team (MDT) recommendation. Synchronous breast and colon cancers pose unique diagnostic and treatment planning challenges. MDT collaboration is crucial for personalized treatment strategies and optimized outcomes in these complex cases.

## 1. Introduction

Multiple primary malignant neoplasms (MPMNs), defined as two or more histologically distinct tumors in one individual [1–5], can be synchronous or metachronous, occurring in 0.7%–11% of patients [4–7]. A SEER database analysis (1988–2007) of 4835 women with breast and colon cancers reported a 3.8% frequency of synchronous cancers [2]. This represents, to our knowledge, the second documented case in Africa, as only one prior case has been formally published in the peer-reviewed literature [8]. BRCA1 and BRCA2 mutations play a significant role in the development of these tumors [7]. The management of synchronous solid tumors is uniquely challenging due to the absence of standardized treatment protocols [6, 9, 10]. While established guidelines exist for individual malignancies, concurrent presentation complicates treatment decisions, particularly regarding surgical timing, feasibility of simultaneous resections, neoadjuvant therapy selection, and follow-up strategies [2, 9]. This report describes the case of a 73-year-old woman in Senegal diagnosed with synchronous invasive ductal carcinoma (IDC) of the breast and adenocarcinoma of the right colon. This case emphasizes the critical role of multidisciplinary team (MDT) collaboration in managing the complex challenges posed by such synchronous cancers and underscores the importance of individualized treatment strategies for optimal patient outcomes.

## 2. Case Presentation

A 73-year-old woman presented with a 5-month history of a right breast mass and 6-kg unintentional weight loss. Menarche was at 12, and menopause at 53. She denied personal or family history of malignancy, abdominal pain, changes in bowel movements, or gastrointestinal bleeding. She also denied smoking, alcohol or illicit drug use, carcinogen exposure, and prior irradiation. On examination, she was vitally stable. A 6 × 5 cm right upper outer quadrant breast mass, adherent to the pectoralis major and skin with an “orange peel” appearance, was noted. Multiple mobile ipsilateral axillary lymph nodes were palpable; no supraclavicular lymphadenopathy was present. The left breast exam and the remainder of the physical exam were unremarkable.

**Figure 1:**
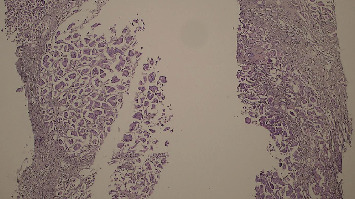
Histopathological examination of the breast biopsy revealing a Grade 2 invasive ductal carcinoma according to the Scarff–Bloom–Richardson grading system.

**Figure 2:**
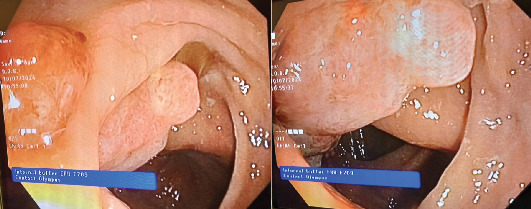
Colonoscopy showing an ulcerated, budding, circumferential, and nonobstructive lesion in the right colon, located distal to the cecum and extending approximately 10 cm in length.

**Figure 3:**
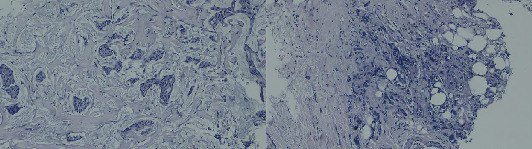
Histopathological examination of the colonic tumor biopsy revealing an invasive, moderately differentiated adenocarcinoma.

Core needle biopsy (Figure 1) confirmed Grade 2 IDC. Immunohistochemistry (IHC) showed estrogen receptor (ER) and progesterone receptor (PR) positivity, HER2-neu negativity, and a 25% Ki-67 index. Staging CT revealed T4cN1M0 breast cancer and incidental right colon wall thickening. Colonoscopy revealed a 10-cm, ulcerated, budding, circumferential, nonobstructive lesion in the right colon (Figure 2). Biopsy confirmed moderately differentiated invasive adenocarcinoma (Figure 3) with cytokeratin 20 (CK20) positivity and CK7 negativity on IHC. Further IHC confirmed the breast lesion as primary IDC (CK7 positive and consistent hormone receptor profile). Pretherapeutic workup including complete blood count, fasting blood sugar, liver and renal chemistries, electrocardiogram, echocardiography, CA 19-9, CEA, CA 15-3, HIV, and acute hepatitis panel was unremarkable.

**Figure 4:**
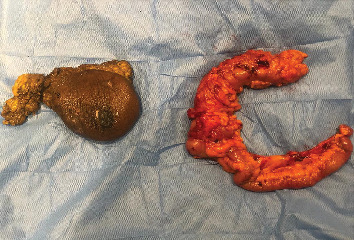
Modified radical mastectomy (left side) and right hemicolectomy (right side) specimens.

The MDT recommended four cycles of neoadjuvant carboplatin and paclitaxel, followed by simultaneous radical mastectomy with axillary clearance and right hemicolectomy (Figure 4). Recovery was uneventful, and she was discharged on Postoperative Day 7. Breast pathology showed residual IDC foci (near complete response), vascular emboli, and 3 of 7 positive lymph nodes (Sataloff TaNc, ypT1N1Mx). Hemicolectomy pathology revealed moderately differentiated mucinous adenocarcinoma; all eight examined lymph nodes were negative (ypT3N1Mx). She received four adjuvant carboplatin/paclitaxel cycles, then external beam radiation (50 Gy in 25 fractions over 5 weeks) to the right chest wall and axilla, followed by anastrozole 1 mg daily. At 9-month follow-up, no local recurrence or distant metastasis was evident.

## 3. Discussion

MPMN refers to two or more distinct primary malignant tumors in one patient [1–3], occurring in 0.73%–11% of patients [5, 10]. MPMN can be synchronous (diagnosed concurrently or within 6 months) or metachronous (diagnosed > 6 months apart) [1, 10]. While metachronous MPMNs are more common (averaging 17% prevalence), synchronous cancers are rarer (3%–5% frequency) [5, 9]. MPMN incidence may be rising due to increased awareness, improved diagnostics, genetic predispositions, environmental exposures, treatment effects, immunosuppression, and longer lifespans.

Our case fulfilled the Warren and Gates criteria for MPMN: (i) histologically malignant tumors, (ii) distinct pathological features for each tumor, (iii) occurrence at different sites/organs (or distinct locations within the same organ), and (iv) exclusion of metastases/recurrences [3].

Synchronous breast and colon cancers are rare (3.8% of patients with both malignancies) [2, 5]. While a second primary malignancy occurs in approximately 3% of breast cancer patients, the link between synchronous breast and colon cancers is debated [1, 4, 9]. Familial predisposition, including mutations like CHEK2⁣^∗^1100delC reported in patients with hereditary breast and colorectal cancer phenotypes, can increase risk [1, 4]. While inherited mutations—such as mismatch repair gene mutations in Lynch syndrome, TP53 gene in Li–Fraumeni syndrome, and APC gene in familial adenomatous polyposis—increase susceptibility to multiple malignancies [4, 5], breast cancer is not typically associated with HNPCC, and our patient did not meet the Amsterdam II criteria for Lynch syndrome. Given the absence of family history, a sporadic germline mutation was considered. The patient and her family received genetic counseling, but financial constraints and lack of local test availability precluded testing.

Reported synchronous breast and colon cancers typically occur in women aged 50–87, consistent with our patient's age [2, 4–6, 8–12]. Most cases involve IDC of the breast [5, 6, 8–10, 13], although invasive lobular carcinoma has been reported [11, 12]. No specific breast cancer histology or IHC profile, or colonic adenocarcinoma site, has been linked to increased synchronous cancer risk.

The second malignancy is often incidentally discovered during staging of the primary tumor [4, 5, 8–10]. In our patient, breast cancer symptoms predominated, and the colonic tumor was found during staging. IHC analysis is crucial for diagnosing synchronous malignancies and excluding mutual metastases. Asaad et al. reported that positivity for CDX and CK20, along with negativity for GCDFP-15 and GATA-3, supports the colonic origin of adenocarcinoma [6]. For breast cancer, the expression of ER, PR, and HER2-neu is commonly analyzed [5, 9]. In cases of MPMN, the positivity of GATA-3 and CK7 confirms breast origin. The IHC profiles in our patient were consistent with synchronous MPMN.

Synchronous neoplasms pose unique treatment challenges. While some prioritize less invasive initial surgeries [2, 10], simultaneous resections are also reported [2]. Our patient underwent simultaneous mastectomy and hemicolectomy after neoadjuvant chemotherapy. The limited retrieved lymph nodes (mastectomy: 7; hemicolectomy: 8) may have impacted staging accuracy. While a more comprehensive dissection would have been ideal, neoadjuvant chemotherapy and surgical technique could have contributed to the low yield.

Due to the lack of established guidelines, treatment strategies must be individualized through MDT review [1, 4]. Our MDT recommended neoadjuvant chemotherapy followed by surgery. The locally advanced tumor stage and incomplete lymph node clearance justified adjuvant chemoradiation. The regimen and duration were selected based on tumor characteristics, drug availability, and the patient's overall performance status.

Synchronous tumor prognosis depends on each malignancy's stage [1, 4]. With effective management, synchronous cancers do not necessarily have worse outcomes than single malignancies [4]. The challenge lies in developing a therapeutic strategy that addresses both malignancies without excessive toxicity, drug interactions, or compromised overall outcomes [4].

## 4. Conclusion

Synchronous breast and colon neoplasms are rare and present significant therapeutic challenges. In the absence of guidelines, MDT collaboration is essential for optimal decision-making. Clinicians should maintain a high index of suspicion for synchronous malignancies in patients with newly diagnosed breast cancer, and colonoscopy should be considered during follow-up.

## Data Availability

The data that support the findings of this study are available on request from the corresponding author. The data are not publicly available due to privacy or ethical restrictions.

## References

[1] Tripodi D., Cannistra’ C., Gagliardi F. (2022). Coincidental or causal? Concurrence of colorectal carcinoma with primary breast cancer. *Digestive Diseases and Sciences*.

[2] Gadiyaram S., Nachiappan M., Thota R. (2022). Metastatic sigmoid colon malignancy with a synchronous carcinoma breast: is cure possible?. *Cureus*.

[3] Hao L., Zhang L., Xu C., Jiang M., Zhu G., Guo J. (2023). Multiple synchronous primary malignant neoplasms: a case report and literature review. *Oncology Letters*.

[4] Abdulla H. A., Almarzooq R., Alrayes A. (2019). Synchronous breast and colon cancer: the importance of multidisciplinary team cancer meetings. *BML Case Reports*.

[5] Bin Saleem M. Y., Albandar M. H., Alfaifi J. A. (2022). Synchronous colon and breast cancers: a case report of multiple primary tumors. *Cureus*.

[6] Asaad A., Barron M., Rasheed N., Idaewor P., Saad Abdalla Al-Zawi A. (2021). The rare diagnosis of synchronous breast and colonic cancers: a case report and review of literature. *Cureus*.

[7] Weissman S., Sebrow J., Gonzalez H. H. (2019). Diagnosis of primary colorectal carcinoma with primary breast cancer: associations or connections?. *Cureus*.

[8] Randriamanovontsoa N., Raherinantenaina F., Ranaivomanana A., Rafaramino F. (2014). Association synchrone d’un cancer du sein et du côlon chez la femme. *Médecine d'Afrique Noire*.

[9] Higgins L., Robertson I., Khan W., Barry K. (2013). Synchronous breast and colon cancer: factors determining treatment strategy. *Case Reports*.

[10] Yetkin G., Celayir F., Akgun I. E., Ucak R. (2017). Synchronous occurrence of primary breast carcinoma and primary colon adenocarcinoma. *Case Reports in Surgery*.

[11] Jafferbhoy S., Paterson H., Fineron P. (2014). Synchronous gist, colon and breast adenocarcinoma with double colonic polyp metastases. *International Journal of Surgery Case Reports*.

[12] Koufopoulos N., Goudeli C., Pigadioti E. (2018). Synchronous colonic adenocarcinoma and metastatic lobular carcinoma in a colectomy specimen: a rare finding. *Cureus*.

[13] Anania G., Santini M., Marzetti A. (2012). Synchronous primary malignant tumors of the breast, caecum and sigma. Case report. *Il Giornale di Chirurgia-Journal of the Italian Surgical Association*.

